# Long noncoding RNA MALAT1 inhibits the apoptosis and autophagy of hepatocellular carcinoma cell by targeting the microRNA-146a/PI3K/Akt/mTOR axis

**DOI:** 10.1186/s12935-020-01231-w

**Published:** 2020-05-13

**Authors:** Ningfu Peng, Jingrong He, Jindu Li, Hao Huang, Weiqiao Huang, Yingyang Liao, Shaoliang Zhu

**Affiliations:** 1 Department of Hepatobiliary Surgery, Guangxi Medical University Cancer Hospital, No. 71, Hedi Road, Qingxiu District, Nanning, 530021 Guangxi China; 2 Department of Clinical Nutrition, Guangxi Medical University Cancer Hospital, Nanning, 530021 Guangxi China

**Keywords:** HCC, lncRNA, miR, PI3K, Apoptosis, Autophagy

## Abstract

**Background:**

Increased long noncoding RNA (lncRNA) expression is characteristic to hepatocellular carcinoma (HCC) and several other neoplasms. The present study aimed to identify the mechanism underlying modulation of HCC development by the lncRNA metastasis-associated lung adenocarcinoma transcript 1 (MALAT1).

**Methods:**

Quantitative real-time polymerase chain reaction was used to determine MALAT1 and microRNA (miR)-146a expression in HCC tissues and cell lines. Western blotting was performed to measure PI3K, Akt, and mTOR levels. Dual-luciferase reporter assay was used to validate the direct targeting and negative regulatory interaction between miR-146a and MALAT1. Cell viability, proliferation, and apoptosis were analyzed using an 3-(4,5-dimethylthiazol-2-yl)-2,5-diphenyl tetrazolium bromide assay, colony formation assay, and flow cytometry, respectively; autophagy was detected based on LC3B expression.

**Results:**

MALAT1 expression was higher in HCC tissues than in normal tissues. MALAT1 upregulation promoted HCC cell proliferation, whereas MALAT1 downregulation promoted HCC apoptosis and autophagy. Moreover, effects of MALAT1 downregulation on HCC cells were abolished by miR-146a inhibition. miR-146a directly targeted the 3′-untranslated region of *PI3K*, and PI3K protein level was clearly decreased upon miR-146a mimic transfection.

**Conclusions:**

MALAT1 may modulate HCC cell proliferation, apoptosis, and autophagy via sponging miR-146a, which regulates HCC progression.

## Background

Hepatocellular carcinoma (HCC) shows frequent recurrence, poor prognosis, and high associated morbidity, thereby posing a threat to public health [1, 2]. Alcoholism, aflatoxin intake, and hepatitis are the etiological factors that increase the risk of HCC development [3, 4]. HCC develops into malignancies through genetic and epigenetic variations [5]. Most patients with HCC are diagnosed at an advanced stage, when most therapies are ineffective. Although surgical resection is accepted as a treatment option and prolongs survival, over two-third of the patients experience recurrence [6]. While molecular mechanisms of HCC have been explored in a few studies, these efforts have not translated into improvement in patient survival. Therefore, there is a clinically unmet need for finding novel therapeutic targets for HCC.

As reported in several studies, increased expression of the long noncoding RNA (lncRNA) metastasis-associated lung adenocarcinoma transcript 1 (MALAT1) is characteristic to human oncogenesis. MALAT1 accelerates tumor metastasis, solid tumor formation, and hematologic malignancy development. Thus, MALAT1 expression is a potential cancer biomarker. Meta-analyses have demonstrated the correlation between MALAT1 overexpression and poor clinical outcomes [7, 8]. MALAT1 overexpression, primarily in HCC [9], leads to disease progression, reduces overall survival [10], and increases the risk of relapse in patients undergoing hepatic transplantation [11]. Evidence suggests that MALAT1 and NEAT1 are hepatocarcinogenesis-associated mutational factors [12].

MicroRNAs (miR), which are ~ 22-nucleotide long, may regulate gene expressions by binding to the complementary 3′-untranslated regions (UTRs) of their target mRNAs [13, 14]. lncRNAs that sponge miRs may regulate gene transcription in HCC [15]. In a previous study, miR-146a-5p enhanced radiation sensitivity of HCC via activation of the DNA repair pathway by the replication protein A3 [16].

The PI3Ks, which were able to be divided into three classes (class IA, class IB, class II and class III), are important kinases regulating cell survival, proliferation and differentiation [17, 18]. The activated PI3K produced the second messenger PIP3 from PIP2 by phosphorylating inositols on the lipid membrane. Then the serine–threonine kinase Akt is recruited to the membrane via binding to PIP3, resulting in its phosphorylation by phosphoinositide-dependent kinases [19]. Once activated, Akt activates downstream signaling effectors to regulate cell survival, proliferation, cell cycle progression, migration and angiogenesis. The PI3K–Akt signaling pathway is widely reported to be involved in the development of HCC, breast cancer, and gastric carcinoma [20–23]. Dysregulation of the PI3K/Akt pathway, which is a prototypic survival pathway, is increasingly implicated in HCC carcinogenesis [24].

However, there is limited knowledge regarding the effects of miR-146a-5p on HCC cell proliferation and interaction of miR-146a-5p with MALAT1 and PI3K/Akt; therefore, the present study aimed to identify the mechanism underlying modulation of HCC development by MALAT1.

## Materials and methods

### Samples and cell lines

Forty people who were pathologically diagnosed with HCC at Guangxi Medical University Cancer Hospital and who underwent tumor resection before any preoperative therapy were included. The subjects comprised 29 males and 11 females, with the average age of 65.4 ± 2.7 years. Twenty-two patients presented with HCC of stage I + II and 18 with HCC of stage III + IV. Twelve patients were recruited as healthy controls, and their normal liver tissues were sampled. This study was approved by the ethics committee of Guangxi Medical University Cancer Hospital, and all patients provided informed consent. The tissue samples were stored at − 80 °C in liquid nitrogen and subsequent RNA extraction (Table 1).

**Table 1:** Association of MALAT1 expression with clinicopathological features of HCC patients (n = 40)

Feature	Relative MALAT1 expression		P value
	Low (n = 11)	High (n = 29)	
Age			
≤ 50	4	4	0.503
> 50	7	25	
Sex			
Male	9	20	0.602
Female	2	9	
AFP (μg/L)			
≤ 20	5	19	0.403
> 20	6	10	
HBV			
Positive	7	13	0.521
Negative	4	16	
Liver cirrhosis			
Yes	8	26	0.063
No	3	3	
Tumor diameter (cm)			
≤ 5	8	7	0.022
> 5	3	22	
TNM stage			
I + II	9	5	0.010
III + IV	2	24	

The human immortal liver cell lines LO2, HepG2, and Huh7 were supplied by the American Type Culture Collection (Manassas, VA). The cell lines were maintained in Dulbecco’s modified Eagle medium (GIBCO^®^, Gaithersburg, MD) containing 10% fetal bovine serum in a humidified incubator at a controlled temperature of 37 °C (5% CO_2_). All cell lines, including the normal hepatocyte cell line, were maintained in the laboratory where the trials were performed.

### Quantitative real-time polymerase chain reaction (qRT-PCR)

qRT-PCR was performed using the TaqMan^®^ MicroRNA Assay kit in accordance with the manufacturer’s instructions. GAPDH and U6 RNA were used as internal controls to normalize variations in total RNA levels. All PCR runs were performed in triplicate. Agarose gel electrophoresis was performed to separate PCR products, and ethidium bromide was adopted as a fluorescent stain for visualization. A comparative threshold cycle method was used for calculating relevant gene expressions, and Ct was the cycle threshold for determining relative RNA level using the 2^−ΔΔCt^ method. The primers sequences were as follows: MALAT1 forward: 5′-AAA GCA AGG TCT CCC CAC AAG-3′ and MALAT1 reverse: 5′-GGT CTG TGC TAG ATC AAA AGG C-3′; GAPDH forward: 5′-GGT GGT CTC CTC TGA CTT CAA CA-3′, and GAPDH reverse: 5′-GTG GTC GTT GAG GGC AAT G-3′; miR-146a forward: 5′-CCG ATG TGT ATC CTC AGC TTT G-3′ and miR-146a reverse: 5′-GCT GAA GAA CTG AAT TTC AGA GGT C-3′; U6 forward: 5′-TGC GGG TGC TCG CTT CGG CAG C-3′ and U6 reverse: 5′-CCA GTG CAG GGT CCG AGG T-3′; PI3K forward: 5′-AAC ACA GAA GAC CAA TAC TC-3′ and PI3K reverse: 5′-TTC GCC ATC TAC CAC TAC-3′.

### Western blotting (WB)

Western blotting was performed as described previously [25]. Briefly, a total protein preparation was first made using the Beyotime complete cell lysis buffer (P0013, Shanghai, China) containing protease (11697498001, Sigma) and phosphatase (5870, CST) inhibitors as essential components. Next, identical quantities of proteins, which were isolated from the total prepared protein subjected to 10% sodium dodecyl sulfate-polyacrylamide gel electrophoresis, were transferred onto Millipore PVDP membranes (IESN07852, Merck). Secondary antibody (ab6721 and ab6728, Abcam) was added to maintain the membranes after they were hybridized with antibodies against enhancers of PI3K (1:500, ab32089, Abcam), Akt (1:1000, ab8805, Abcam), mTOR (1:2000, ab2732, Abcam), and β-actin (1:5000, ab8226, Abcam). The bands were scanned using a Bio-Rad ChemiDocXRS + System.

### Construction and transfection of lncRNA and miR siRNA

Ribobio (Guangzhou, China) synthesized short-interfering RNAs (siRNAs) (siMALAT1 and siNC) and provided miR-146a inhibitors/mimics. Using Lipofectamine 2000 (Invitrogen), 2 × 10^6^ cells were transfected with 4 μg miR-146a inhibitors/mimics, corresponding negative control (NC) primers, or 8 μg siRNAs for 48 h before subsequent experiments and test were performed. Their sequences were as follows: si-MALAT1, 5′-GCA AAU GAA AGC UAC CAA UTT-3′; si-NC, 5′-UUC UCC GAA CGU GUC ACG UTT-3′; miR-146a mimic, 5′-CCU CUG AAA UUC AGU UCU UCA G-3′; NC mimic, 5′-UCA CAA CCU CCU AGA AAG AGU AGA-3′; miR-146a inhibitor, 5′-CCU CUG AAA UUC AGU UCU UCA G-3′; NC inhibitor, 5′-UCA CAA CCU CCU AGA AAG AGU AGA-3′.

### 3-(4,5-Dimethylthiazol-2-yl)-2,5-diphenyl tetrazolium bromide (MTT) assay

For assessing cell viability, 2 × 10^6^ cells in a 24-well plate were treated with 20 μL MTT (0.5 mg/mL) for 20 min. Next, dimethyl sulfoxide (150 μL) was added into each well, and the cells were mixed for 10 min with orbital shaking to dissolve formazan dye. Finally, absorbance at 490 nm was measured using the Infinite M200 microplate reader.

### Colony formation assay

A total of 1 × 10^5^ cell were spread onto an 8-mm 0.4% top agar layer and then transferred onto a 0.5-mm 0.5% bottom agar beds in 12-well plates. After 14 days, four regions were randomly selected from each plate for colony counting.

### Apoptosis analysis

Apoptotic cells were characterized using Annexin V and propidium iodide (PI) staining with flow cytometry [26]. Apoptotic cells were stained after 48 h of transfection using the Annexin V/PI apoptosis detection kit (KGA-1012, KeyGEN, Nangjing, China). A total of 2 × 10^5^ cells were suspended in 50 μL binding buffer and treated as follows: 5 μL/sample PI for 15 min and 1 μL/sample Annexin V and 450 μL/sample binding buffer for 15 min. Positively stained cells were detected using the BD FACS Canto II Flow Cytometer (Becton–Dickinson, Franklin Lakes, NJ, USA).

### TargetScan prediction

The prediction algorithms TargetScan and LncTar were used to identify miR-146a and MALAT1 targets. On the website (http://www.targetscan.org) or LncTar (http://www.cuilab.cn), predictions are listed according to their efficacy of target prediction [27]. As an alternative, predictions are also ranked by their probability of conserved targeting [28].

### Dual-luciferase reporter assay (DLRA)

DLRA was used to investigate miR-146a target genes using pmiR-RB-Report™-PI3K/MALAT1-3′-UTR-mutant (MU) and PI3K/MALAT1-3′-UTR-wild-type (WT). Cells transfected with miR-146a mimic/NC mimic and pmiR-RB-Report™ were cultured in prepared medium for 24 h. Next, firefly and *Renilla* luciferase activities were determined.

### Statistical analysis

Results are expressed as mean ± standard deviation (SD). Two-tailed Student’s t test and ANOVA with post hoc Tukey test were used for between-group and inter-group comparisons, respectively. Differences were considered significant at P < 0.05.

## Results

### HCC tissues and cells showed elevated MALAT1 expression

**Fig. 1:**
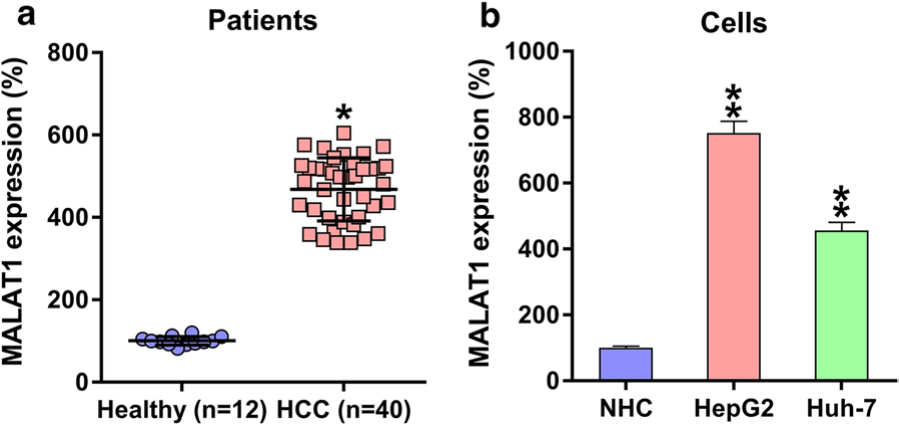
MALAT1 expression in HCC samples/cell lines. **a** Q-PCR was used to measure the MALAT1 expression in HCC specimens obtained from subjects with HCC (n = 40) and from specimens obtained from healthy volunteers (n = 12). **b** MALAT1 expression in HepG2/Huh-7 cell lines and in healthy human hepatocytes. Results are expressed as mean ± SD. *P < 0.05, **P < 0.01, in comparison with the indicated group

qRT-PCR was used to measure MALAT1 expression in HCC tumors. As shown in Fig. 1a, MALAT1 expression was upregulated in HCC tumor samples compared with that in normal tissues. In addition, two HCC cell lines, HepG2 and Huh-7, showed higher MALAT1 expression than the normal human hepatic cells (Fig. 1b).

### MALAT1 silencing suppressed HCC cell multiplication

**Fig. 2:**
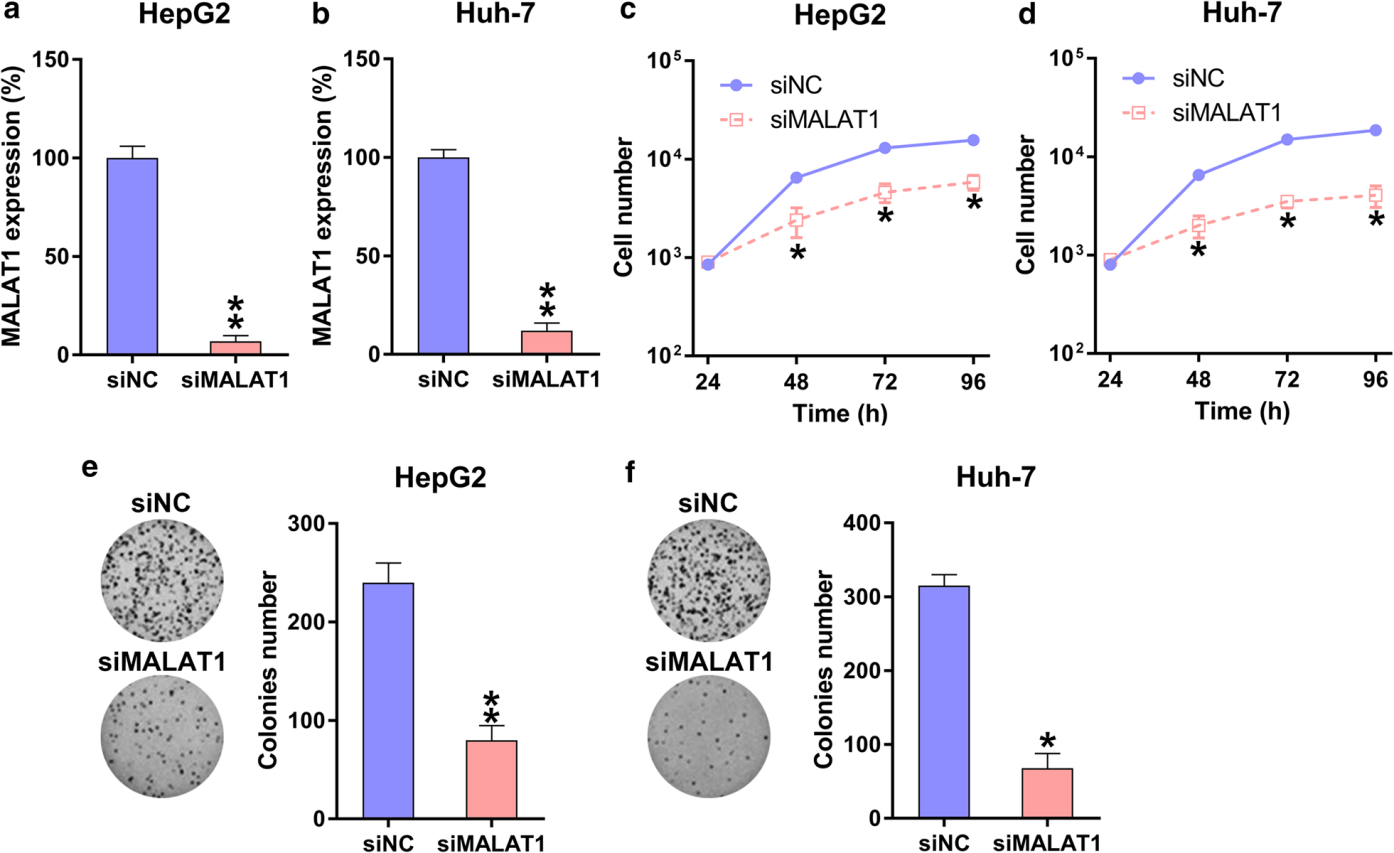
Role of MALAT1 silencing in HCC cell multiplication. **a**, **b** Q-PCR was used to measure MALAT1 expression in HepG2 and Huh-7 cells transfected with siMALAT1 or siNC for 48 h. **c**, **d** Multiplication rates of the HepG2 and Huh-7 cells at 24, 48, or 72 h after transfection were tested using the MTT assay. **e**, **f** A soft-agar colony formation assay was performed for HepG2 and Huh-7 cells that were transfected with siMALAT1 or siNC at 48 h. The data were described as mean ± SD. *P < 0.05, **P < 0.01, as compared with the indicated group

For testing the role of MALAT1 in the viability of two HCC cell lines, HepG2 and Huh-7, MALAT1 was first silenced. When transfected with the siMALAT1 or siNC vector, cells showed significantly reduced MALAT1 expression (Fig. 2a, b). Using MTT assay, siMALAT1-transfected HepG2 cells and Huh-7 cells showed significantly decreased proliferation rates at 24–72 h compared with siNC-transfected cells (Fig. 2c, d). Colony formation assay further confirmed that the growth of HCC cells was significantly reduced upon MALAT1 silencing (Fig. 2e, f).

### MALAT1 silencing induced HCC cell apoptosis and autophagy

**Fig. 3:**
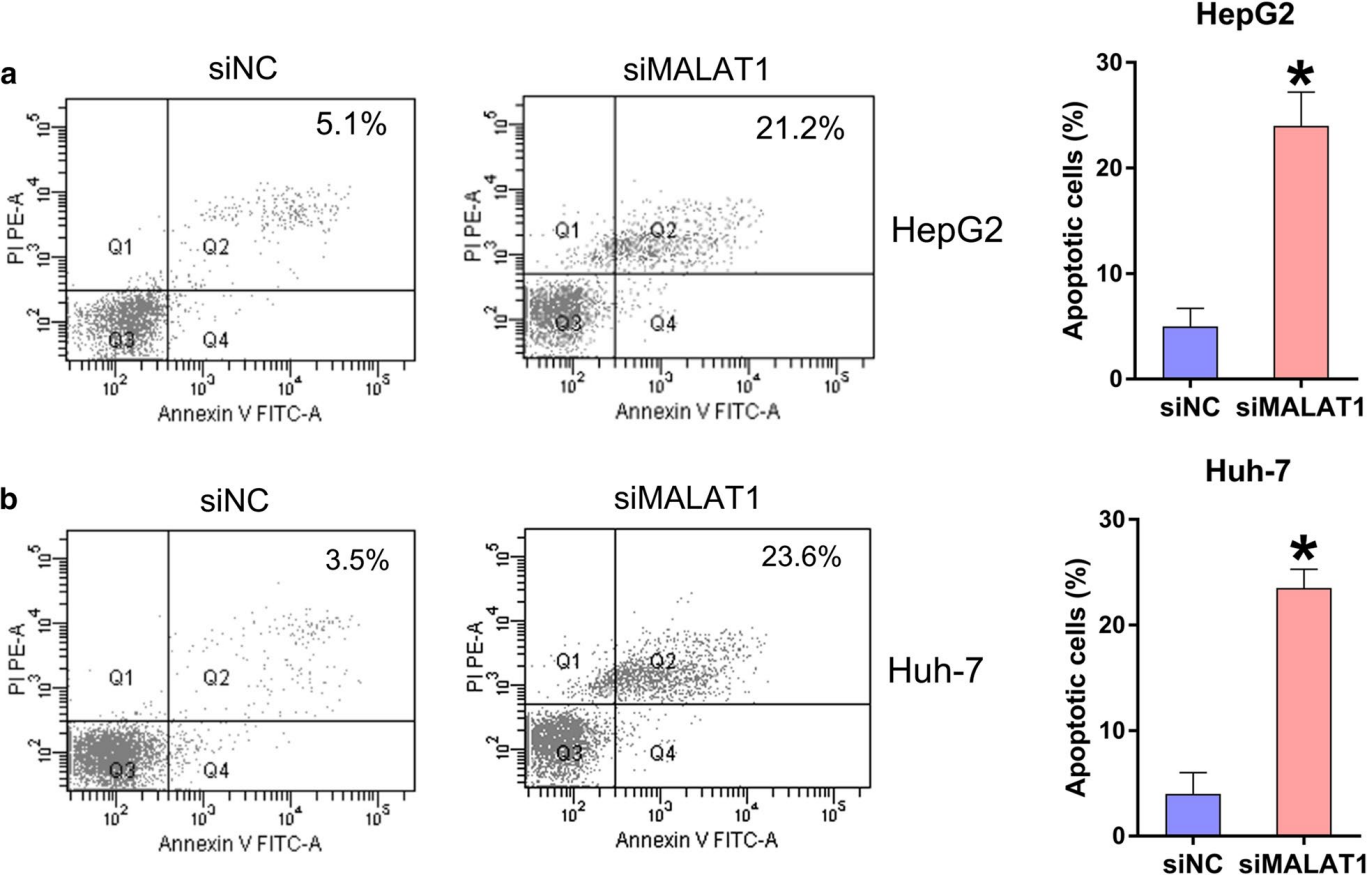
Role of MALAT1 silencing in HCC cell death. HepG2 and Huh-7 cells were transfected with siMALAT1 or siNC for 48 h. **a**, **b** An Annexin V-FITC/PI for FC assay was performed to detect how many apoptotic HepG2 and Huh-7 cells were transfected with siMALAT1 or siNC. The UR quadrant of each FC plot illustrated apoptotic cells. Data were shown as mean ± SD. *P < 0.05, in comparison with the indicated group

Since MALAT1 silencing reduced HepG2 and Huh-7 cell viability, we hypothesized that MALAT1 regulates HCC cell death via apoptosis and autophagy. Annexin V-FITC/PI flow cytometry revealed more conspicuous apoptosis in both siMALAT1-transfected HCC cell lines compared with that in NC-transfected cell lines (Fig. 3a, b), indicating that MALAT1 depletion induced HCC cell apoptosis.

**Fig. 4:**
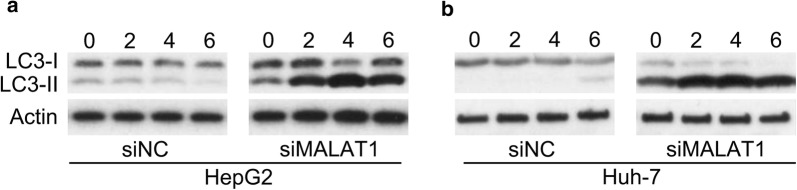
Role of MALAT1 silencing in HCC cell autophagy. HepG2 and Huh-7 cells were transfected with siMALAT1 or siNC for 48 h. **a**, **b** WB was adopted herein to detect the levels of LC3B I and II at 0–6 h post 50 nM bafilomycin A1 administration, in HCC with transfection of siMALAT1 or siNC for 48 h

To measure the maturation of autophagic vacuoles, HCC cells were treated with bafilomycin A1 to inhibit fusion between autophagosomes and lysosomes and accumulate LC3B [29]. MALAT1 silencing induced autophagy of HepG2 and Huh-7 cells, as evidenced by increased LC3B transformation and processing (increased LC3B II levels) following bafilomycin A1 treatment in a time-dependent manner (Fig. 4a, b).

### MALAT1 directly targets miR-146a

**Fig. 5:**
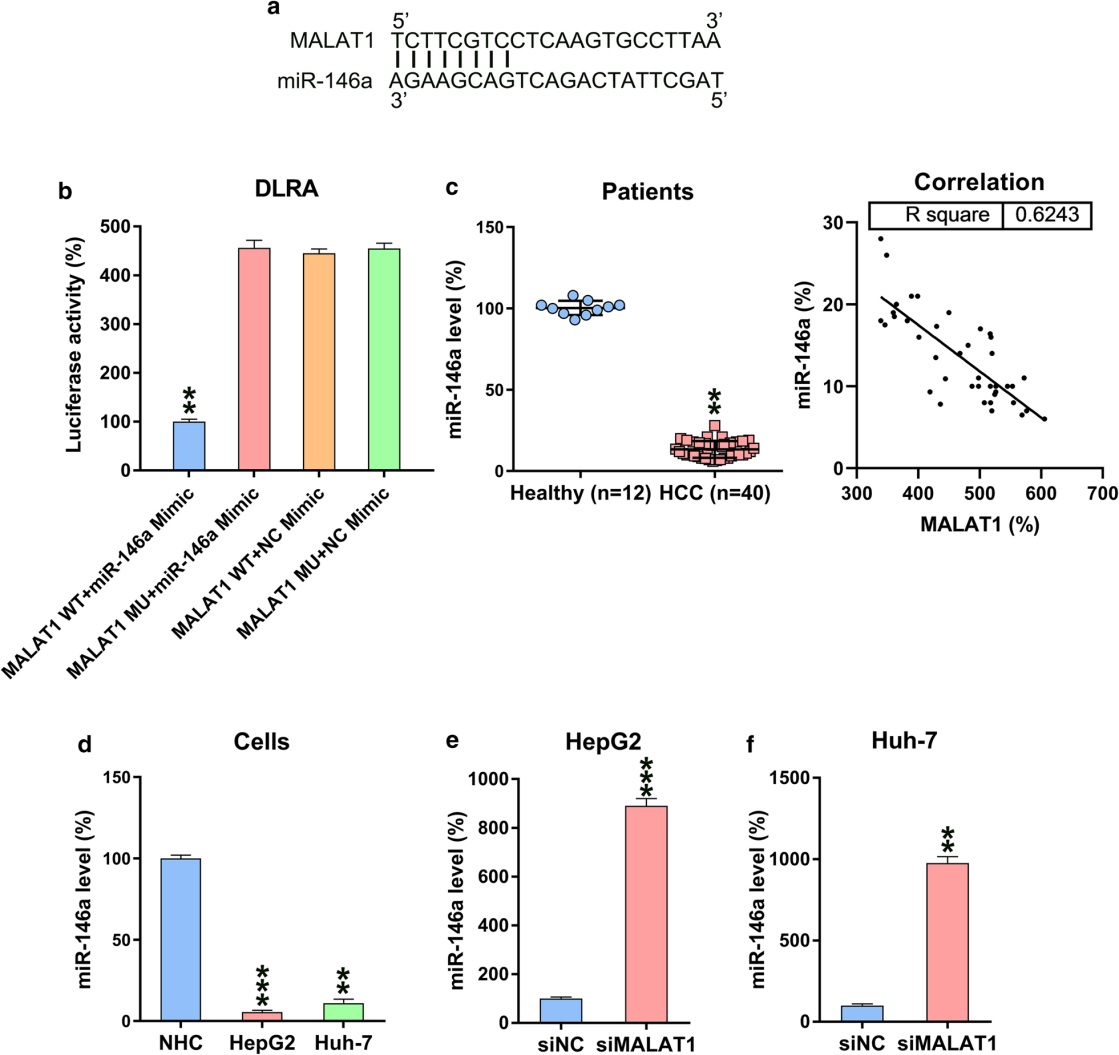
MALAT1 targets miR-146a. **a** As shown in bioinformatic analysis, miR-146a has a binding site in MALAT1. **b** DLRA was subsequent to simultaneous transfection of a luciferase reporter with a wild-type or mutant from MALAT1, and miR-146a mimics/NC into HEK293T cells. **c** Q-PCR analysis illustrated downregulated miR-146a levels in HCC specimens from patients (n = 40) and healthy volunteers (n = 12). Correlation analysis of tumor tissues from 40 HCC cases between MALAT1 and miR-146a expression (P < 0.001). **d** Q-PCR displayed how miR-146a was expressed in HepG2, Huh-7, and normal human hepatic cells. **e**, **f** miR-146a levels in HepG2/Huh-7 cells, which underwent siMALAT1 and siNC transfection, were detected using Q-PCR. Data are shown as mean ± SD. *P < 0.05, **P < 0.01, and ***P < 0.001, when in contrast with the indicated group

Bioinformatic analyses showed that MALAT1 targets miR-146a (Fig. 5a). DLRA was performed to determine direct binding between miR-146a and MALAT1 (Fig. 5b). HEK293T cells showed ~ 75% reduced luciferase activity upon transfection with an miR-146a mimic in which WT MALAT1 was fused with an miR-146a mimic. miR-146a expression was reduced in patients with HCC but not in healthy controls. MALAT1 and miR-146a expressions in HCC tumor tissues were negatively correlated (P < 0.001) (Fig. 5c). Similarly, miR-146a expression was reduced in HepG2 and Huh-7 cells but not in normal human hepatic cells (Fig. 5d). Moreover, MALAT1 silencing increased miR-146a expression in siMALAT1- or siNC-transfected HepG2 and Huh-7 HCC cells (Fig. 5e, f).

### miR-146a inhibitor reverses the effects of MALAT1 silencing in HCC cells

**Fig. 6:**
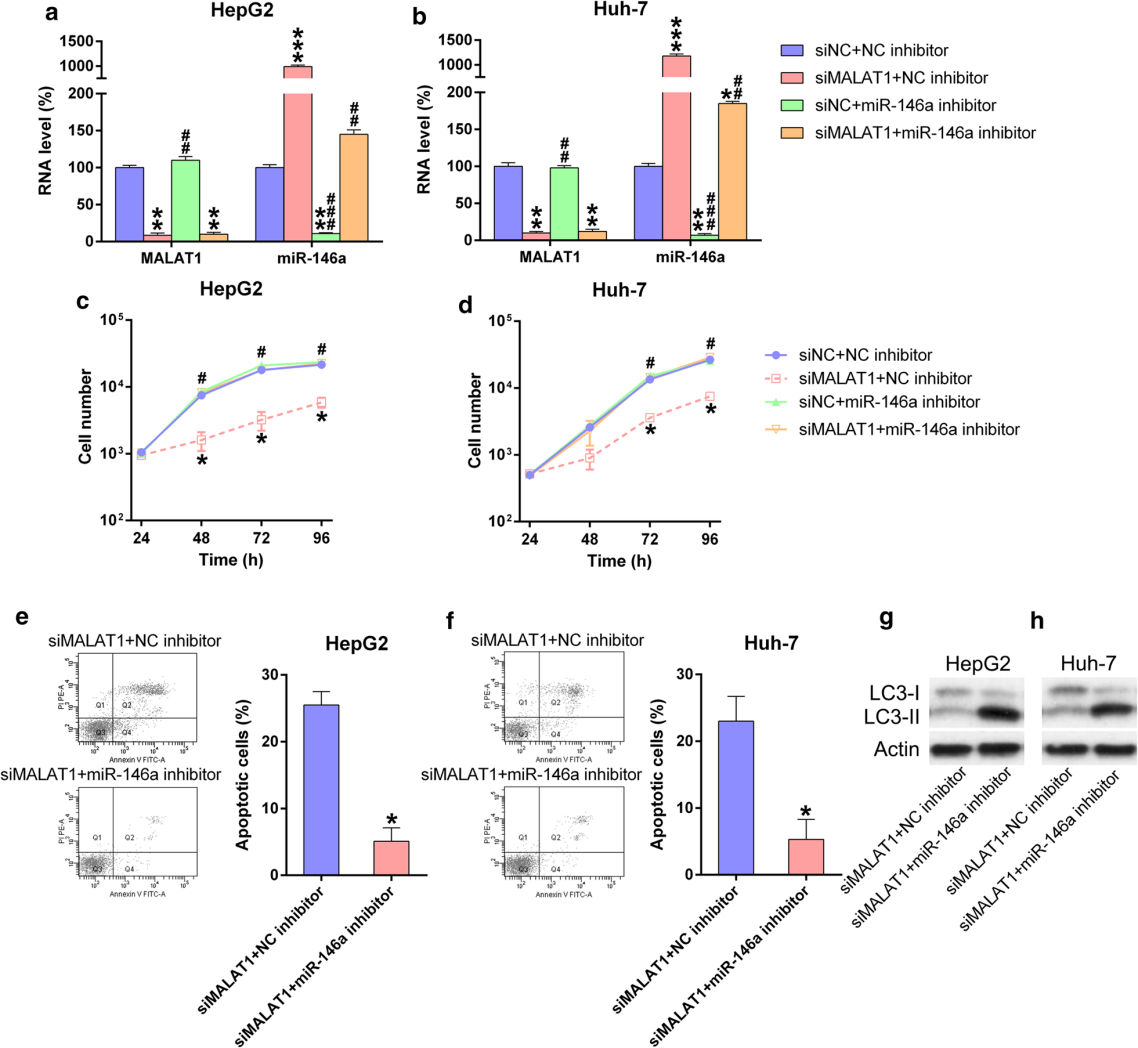
miR-146a inhibitor offset function of MALAT1 in properties of HCC cell lines. HCC cells underwent simultaneous transfection of siMALAT1/siNC and/or miR-146a inhibitor/NC inhibitor for 48 h. **a**, **b** Q-PCR analysis measured the levels of MALAT1 and miR-146a expression in each group. **c**, **d** MTT assay showed the proliferation rate of HCC cell lines during 24–72 h after different transfections. **e**, **f** FC analysis showed the cell number of apoptosis in HepG2 and Huh-7 cells when treated in different ways. Data were shown as mean ± SD. *P < 0.05, **P < 0.01, ***P < 0.001, in comparison with the siNC + NC inhibitor group; ^#^P < 0.05, ^##^P < 0.01, ^###^P < 0.001, in comparison with siMALAT1 + NC inhibitor group

We hypothesized that miR-146a is involved in MALAT1-mediated effects on HCC cells. When simultaneously transfected with siMALAT1/siNC with/without miR-146a/NC inhibitor, miR-146a was significantly upregulated in the siMALAT1 groups and decreased in siMALAT1 + miR-146a inhibitor group. Meanwhile, MALAT1 expression was not affected by miR-146a (Fig. 6a, b). Subsequent MTT assay confirmed the role of miR-146a in regulating cell proliferation. Transfection of the miR-146a inhibitor abolished the inhibitory effects of siMALAT1 on HCC cell proliferation (Fig. 6c, d). Moreover, flow cytometry data indicated that miR-146a inhibitor suppressed the effects of siMALAT1 on cell apoptosis, as evidenced by decreased apoptotic cell number (Fig. 6e, f). WB revealed that miR-146a transfection reduced LC3B-II level in HCC cells following MALAT1 silencing (Fig. 6g, h). Therefore, these results suggest that MALAT1 regulated HCC cell proliferation via miR-146a.

### MiR-146a targeted the 3′-UTRs of PI3K

**Fig. 7:**
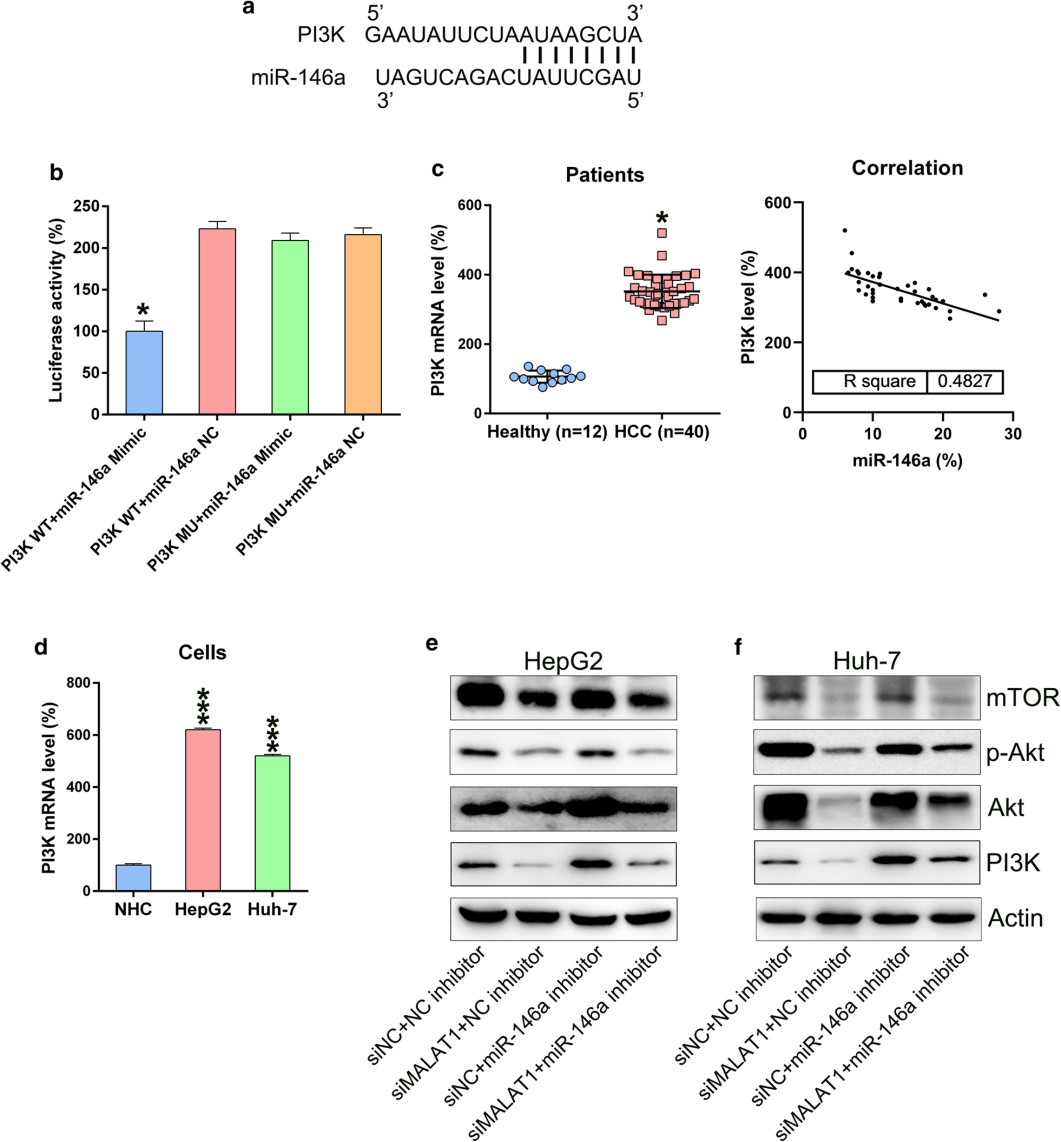
miR-146a targeted the PI3K mRNA 3′-UTR. **a** Bioinformatics research showed a binding site of miR-146a in the PI3K 3′-UTR. **b** DLRA was followed by co-transfecting cells with a luciferase reporter alternatively encompassing a wild-type or mutant PI3K mRNA and a miR-146a mimic into chondrocytes. **c** Q-PCR measured PI3K mRNA expression in patients with HCC (n = 40) and in healthy individuals (n = 12). Correlation analysis of tumor tissues from 40 HCC cases between miR-146a and PI3K mRNA expression (P < 0.001). **d** MALAT1 expression was measured for HepG2 and Huh-7 cell lines and normal human hepatocytes. **e**, **f** HCC cells undergoing transfection by a siMALAT1, siNC, with or without miR-146a inhibitor, or NC inhibitor for 48 h. WB was performed to detect PI3K expression in all groups. The results were shown as mean ± SD. *P < 0.05, ***P < 0.001, in comparison with the indicated group

As shown in Fig. 7a, the 3′-UTRs of PI3K, which is a widely recognized apoptosis and autophagy modulator, may be a target of miR-146a [30]. DLRA was used to investigate the direct binding of miR-146a to the 3′-UTRs of PI3K (Fig. 7b). The findings revealed that miR-146a mimic transfection suppressed the activity of luciferase, which was fused with the 3′-UTR of PI3K, by 50%. Moreover, WB demonstrated that the HCC tissues and cell lines showed increased PI3K levels compared with normal tissues and cells. Further statistical analysis showed that miR-146a and PI3K mRNA expressions in HCC tumor tissues were negatively related (P < 0.001) (Fig. 7c, d). For further confirmation of the triadic relation among MALAT1, miR-146a, and PI3K levels, miR-146a expression in HCC cells was inhibited via siMALAT1 transfection. MALAT1 silencing downregulated miR-146a expression but upregulated PI3K expression, thus affecting the phosphorylation of downstream Akt and mTOR (Fig. 7e, f). These results indicate that miR-146a expression was negatively regulated activation of the PI3K/Akt/mTOR axis.

## Discussion

lncRNA expression is an essential modulator of malignancy, which has been linked to HCC progression. Thorough elucidation of the function and mechanism of lncRNAs may provide insights into HCC diagnosis and treatment. As acknowledged in previous reports, HULC, SRA1, and several other lncRNAs, including DiGeorge syndrome critical region gene 5 (DGCR5) [31–33], are closely involved in HCC. Deregulated lncRNAs are also common in HCC. A previous in vivo study has demonstrated that MALAT1-targeted lentiviral shRNA inhibited tumor ontogenesis in heterograft models of HCC cells transformed by arsenite, enhancing the effects of MALAT1 on carcinogen-induced oncogenesis [10]. Wang et al. [34] revealed a novel system that upregulated MALAT1 expression in HCC via increases in YAP1 expression at both the transcriptional and post-transcriptional levels. Moreover, Wu et al. reported that MALAT1 and HULC-1 are excessively expressed in HCC and show functional, reciprocal actions; they proposed that the reciprocal action induced telomere repeat-binding factor 2 (TRF2) telomerase activity, thereby promoting liver cancer stem cell development [35]. Studies of the mechanisms of HepG2 cells demonstrated that MALAT1 released Sirt1 and increased deacetylase enzymatic activity by competing with Sirt1 deacetylase for binding to DBC1; this deacetylated p53 and hindered its function as a transactivator of the targeted pro-apoptotic genes, leading to uncontrolled cell growth [36]. The human HCC specimens and the two cell lines used in the present study showed MALAT1 upregulation. The role of MALAT1 in regulating HCC cell proliferation and viability was further investigated. Our findings indicated that MALAT1 silencing impaired the proliferation and viability of HCC cells through induced apoptosis and autophagy.

MiR-146a is a well-known miR used in reducing inflammation, which is pivotal in furthering the M2-like phenotype as it is often dysregulated in HCC [37]. miR-146a downregulation has previously been linked to HCC. Various bioinformatic platforms have predicted of 251 genes as potential targets of miR-146a. Among these, 104 are regarded as gene overlaps between HCC and miR-146a. RAC1 is involved in four highly enriched overlapping pathways, making it the most overlapped gene related to miR-146a [38]. Luo et al. [16] confirmed that the properties of HCC cells covered miR-146a overexpression, which inhibited cell proliferation while promoting radiosensitivity and apoptosis. miR-146a overexpression inhibited HCC cell proliferation and invasion. Furthermore, TRAF6 was directly targeted by miR-146a and weakened the effect of miR-146a on HepG2 and SMMC7721 cell proliferation and invasion [39]. Another study demonstrated that DNA methylation reduced miR-146a promoter expression and promoted liver cancer cell migration. miR-146a restoration upregulated APC and thereby suppressed VEGF expression to inhibit HCC cell invasion and migration [40]. Although numerous studies have confirmed the involvement of miR-146a in HCC multiplication, metastasis, and radiosensitivity, the mechanism underlying the suppression of cell proliferation remains unknown. According to the findings of the present study, reduced miR-146a expression in HCC cells increases PI3K expression. Moreover, miR-146a was associated with MALAT1-mediated apoptosis and autophagy. These findings advance our understanding of the intracellular signaling pathways of miR-146a.

The tumor suppressing role of miR-146a and oncogenic role MALAT1 as well as their negative correlation have been demonstrated in HCC and other solid tumors. Previous studies have demonstrated that (1) miR-146a suppresses HCC [39, 40]; (2) MALAT1 downregulates miR-146b-5p in HCC [41]; (3) MALAT1 promotes HCC [42, 43]; and (4) MALAT1 targets miR-146a in acute kidney and lung injury [44, 45]. However, the present study explored the link among HCC, MALAT1, and miR-146a for the first time. Moreover, our study demonstrated the involvement of MALAT1/miR-146a cross-talk in regulating the PI3K/Akt/mTOR axis. The role of PI3K–Akt signaling pathway is well known in the development of multiple cancers, such as HCC, breast cancer, and gastric carcinoma [20–23]. So far, the cellular mechanisms underlying such a widespread activation of the PI3K/Akt pathway in HCC is not fully understood. Zhou et al. demonstrated that the PI3K/Akt pathway is more significantly activated in high-grade HCC tumors and is associated with the poor prognosis in HCC patients. They also suggested that Akt phosphorylation were strongly associated with poor prognosis and poor overall survival [46]. Sahin et al. [47] showed that phosphorylation of mTOR and the expression of its downstream effector, p70S6k, are upregulated in 45% of HCC. Our results suggest that miR-146a inhibits expression of PI3K, Akt, and mTOR, as well as Akt phosphorylation to negatively modulate the PI3K/Akt/mTOR pathway in HCC cell lines. Furthermore, endogenous PI3K was significantly unregulated in HepG2 and Huh-7 HCC cells. However, introduction of exogenous miR-146a in HCC cells dramatically decreased the transduction of the PI3K/Akt/mTOR pathway. These findings are supported by previous reports of upregulation of precancerous features of miR-146a and miR-146b in the thymus gland of T cell-specific PI3K-deficient mice [48], and was consistent with previous report about the activation of PI3K/Akt/mTOR pathway in HCC [24].

## Conclusion

We explored the roles of MALAT1–miR-146a in the pathogenesis of HCC. Consistent with previous studies, we confirmed that MALAT1 and miR-146a serve as transforming genes and tumor inhibitors in HCC neoplasia and progression, respectively. MALAT1 silencing and miR-146a upregulation inhibited tumor properties of HCC cell lines, indicating their potential as therapeutic targets for HCC.

## Data Availability

Not applicable.

## Abbreviations

lncRNA: Long noncoding RNA
HCC: Hepatocellular carcinoma
MALAT1: Metastasis-associated lung adenocarcinoma transcript 1
miR: MicroRNA
qRT-PCR: Quantitative real-time polymerase chain reaction
siRNAs: Short-interfering RNAs
NC: Negative control
MU: Mutant
WT: Wild-type
SD: Standard deviation
FC: Flow cytometry
DGCR5: DiGeorge syndrome critical region gene 5
TRF2: Telomere repeat-binding factor 2
DLRA: Dual-luciferase reporter assay
